# “Entrenched practices and other biases”: unpacking the historical, economic, professional, and social resistance to de-implementation

**DOI:** 10.1186/s13012-015-0211-7

**Published:** 2015-02-13

**Authors:** Theresa Montini, Ian D Graham

**Affiliations:** The Sophie Davis School of Biomedical Education, City University of New York, 160 Convent Avenue, New York, NY 10031 USA; School of Epidemiology, Public Health and Preventive Medicine, University of Ottawa, 451 Smyth Road, Ottawa, Ontario K1H 8M5 Canada

**Keywords:** De-implementation, Social science, Evidence-based medicine

## Abstract

**Background:**

In their article on “Evidence-based de-implementation for contradicted, unproven, and aspiring healthcare practices,” Prasad and Ioannidis (IS 9:1, 2014) referred to extra-scientific “entrenched practices and other biases” that hinder evidence-based de-implementation.

**Discussion:**

Using the case example of the de-implementation of radical mastectomy, we disaggregated “entrenched practices and other biases” and analyzed the historical, economic, professional, and social forces that presented resistance to de-implementation. We found that these extra-scientific factors operated to sustain a commitment to radical mastectomy, even after the evidence slated the procedure for de-implementation, because the factors holding radical mastectomy in place were beyond the control of individual clinicians.

**Summary:**

We propose to expand de-implementation theory through the inclusion of extra-scientific factors. If the outcome to which we aim is appropriate and timely de-implementation, social scientific analysis will illuminate the context within which the healthcare practitioner practices and, in doing so, facilitate de-implementation by pointing to avenues that lead to systems change. The implications of our analysis lead us to contend that intervening in the broader context in which clinicians work—the social, political, and economic realms—rather than focusing on healthcare professionals’ behavior, may indeed be a fruitful approach to effect change.

## Background

In a perfect world, all medical decisions would be based on evidence. While a lofty ideal to which most clinicians aspire, the context of medical practice makes that goal difficult to realize. On the one hand, there are tensions between clinical scientists and clinical practitioners, problems in the translation of research findings, and difficulties in the timing and sequencing of developing, assessing, adopting, and abandoning clinical practice [1-6]. On the other hand, there are conflicts within science, “evidence wars” [7] that weaken the power and influence of the evidence on healthcare practices, and much work that can be characterized as cleaning up the evidence base so that it is strong and pure enough to maximize impact (AGREE Project, Cochrane Collaboration, EQUATOR Network, GRADE Working Group). Narrowing the gap between research and practice is not simply a matter of convincing knowledgeable and conscientious practitioners to do the right thing, but rather acknowledging that clinicians’ practice patterns are located within a social structural context so that even the strongest evidence’s influence is mitigated by the conditions of the context in which health care is practiced.

In their article on “Evidence-based de-implementation for contradicted, unproven, and aspiring healthcare practices,” Prasad and Ioannidis call these factors “entrenched practices and other biases” [7]. We propose to disaggregate “entrenched practices and other biases.” Following the lead of Prasad and Ioannidis [7], we delineate the extra-medical influences of historical, economic, political, and social contexts that should be considered in de-implementation and illustrate using the case example of the de-implementation of radical mastectomy.

## Discussion

### Historical context

Some drugs, devices, and procedures have a long and deep practice history. They are entrenched in organizational infrastructure, and that inertia makes de-implementation of a routinized procedure particularly difficult. The case of radical mastectomy illustrates historical entrenchment.

Radical mastectomy was developed as a treatment for breast disease at the end of the 1800s, when women presented with tumors that were reported to be up to 5 in. in diameter [8,9] that hemorrhaged, were infected by fungus, emitted exhausting discharges, caused much pain, and metastasized [10]. Halsted developed his radical technique—excision of the breast, pectoral (chest) muscles, axillary lymph nodes, and associated skin and subcutaneous tissue—in response to these conditions.

Prior to the late 19th century, cancer was believed to be a blood disorder, not amenable to surgery [11]. That view gave way to the theory of cancer as a local disease. Physicians believed that once the breast succumbed to the disease, it was diseased *in toto* [12], making it necessary to remove the complete breast. Next, they theorized that breast cancer spread via the axilla, so surgeons began removing the axillary nodes, whether or not they evidenced disease [13]. Acceptance of the theory of local disease with centrifugal spread encouraged surgeons to operate early, without waiting for the traditional sign of ulceration of the tumor to make the diagnosis of cancer.

In line with the theory of breast cancer as a local disease that spread centrifugally, Halsted [9] and Meyer [14] contended that cancer spread from the breast and axilla to the pectoral muscles, and therefore, they too should be removed. Halsted [9] also believed that cancer could be spread by cutting through diseased tissues, either because cancer cells were liberated or because a contaminated scalpel would infect new sites. This led to the technique of cutting a wide margin of healthy tissue along with the diseased portion of the breast and removing the entire breast and all associated tissue in one piece at once, akin to amputating a limb.

Halsted’s version of mastectomy was distinguished from that of his colleagues by his insistence on the routine performance of the procedure for the removal of the breast exactly as he had defined it. The glands and tissues specified by Halsted were no longer to be removed or not removed at the discretion of the individual surgeon determining their approach on a case-by-case basis; they were to be removed by him on a regular basis. The Halsted approach signaled the standardization of surgical treatment for breast cancer. Radical mastectomy became the unvarying response to every set of symptoms and every medical history ([15]: page 64), and until 1968, the number of radical mastectomies in the USA increased each year.

**Figure 1:**
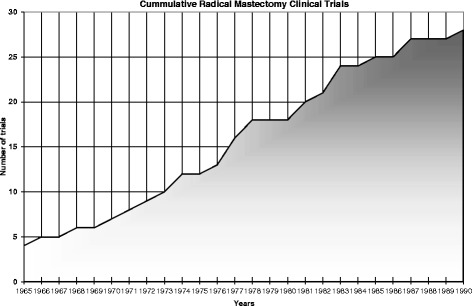
**Cumulative radical mastectomy clinical trials.**

As early as 1941 in Scotland, McWhirter began treating his patients with simple mastectomy and radiation therapy [16,17]. Influenced by McWhirter, an American surgeon, George Crile Jr., began experimenting with lesser surgery and radiation treatment. Crile published his first clinical report in 1961 in the *Annals of Surgery* [18]. By 1965, there were four clinical reports or trials published in the literature. Figure 1 shows the accumulation of the English language evidence base comparing radical mastectomy to lesser procedures up to the 1990 US National Institutes of Health Consensus Development Conference on the treatment of early-stage breast cancer [19]. Of note is that all of the trials except for one, a 1972 clinical trial by Atkins et al. [20], found the lesser surgery to be equal to or better than radical mastectomy or found that lesser surgery with adjuvant therapy was superior to radical mastectomy. Results from the first US clinical trial of postoperative radiation were published in 1970. The number of clinical trials continued to grow, and pooled analysis [21] and meta-analysis [22] confirmed that lumpectomy (breast-conserving surgery to remove a tumor (lump) in a breast and a small amount of normal tissue around it [23]) plus radiation was just as effective as mastectomy.

**Figure 2:**
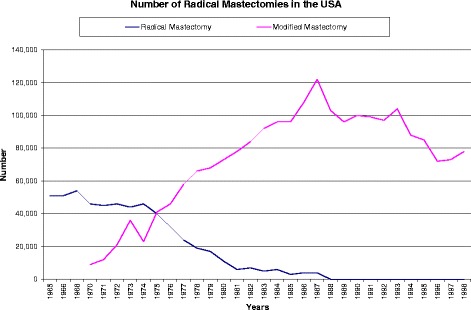
**Number of radical mastectomies in the USA.**

Radical mastectomy had a long reign as the treatment of choice for breast cancer. However, its de-implementation was eased because practitioners did not necessarily have to completely abandon the procedure; they had something they could do instead—modified radical mastectomy (surgery in which the breast, most or all of the lymph nodes under the arm, and the lining over the chest muscles are removed [23]). Figure 2 shows that as radical mastectomy declined, modified radical mastectomy increased in the USA. Therefore, de-implementation of radical mastectomy was facilitated by the development of radiotherapy as adjuvant to lesser surgery and mammography to detect breast cancer at an earlier stage when amenable to treatment with lesser surgery.

The historical view of breast cancer also reveals how worldviews, theories championed by specialists, and individual clinicians’ beliefs about causal mechanisms of disease mesh with practice decisions. When breast cancer was thought to be a blood disorder, surgery was not a viable option. When the paradigm shifted to view breast cancer as a local disease, surgery became the primary option and was used to support and justify the Halsted approach. Others have also noted how clinicians’ beliefs about the body, pathophysiology, disease, and treatments can support change (both adoption and de-implementation) and inhibit change when it runs counter to the current worldview [24]. More recently, Gabbay and le May have highlighted the role of mindlines (collectively reinforced, internalized, tacit guidelines) in professional socialization [2], part of which includes clinicians’ beliefs and worldviews. What is often difficult to tease apart, however, is whether worldviews/beliefs lead to practice change or whether changes in practice catalyze changes in worldviews to justify what they are proposing (sometimes referred to as persuasive knowledge use). In any case, paying attention to the history of the evolution of clinicians’ worldviews and beliefs may provide important insight into the de-implementation process.

### Economic context

Some drugs, devices, and procedures have a complex financial structure that can pit physicians, hospitals, the pharmaceutical industry, and device manufacturers against each other. If de-implementation has financial consequences for a sector that is powerful and profiting, economic entrenchment may be a barrier. The case of radical mastectomy illustrates economic forces of resistance to de-implementation.

In fee-for-service payment systems, the more a provider does, the more profit is generated. Therefore, the de-implementation of radical mastectomy in the USA was up against the desire and need to maintain well-established revenue streams. In 1986, Muñoz and colleagues compared the cost of lumpectomy versus mastectomy for stage I or stage II breast cancer at Long Island Jewish Medical Center [25]. Hospital inpatient and surgeon fees were higher for mastectomy compared to lumpectomy, so one would assume that mastectomy would be the favored option. However, after a lumpectomy, the hospital charged for outpatient radiotherapy, and the services of a physician radiotherapist were also reimbursed. Therefore, when considering who is profiting financially from the radical mastectomy slated for de-implementation, surgeons would lose financially, but hospitals and radiologists would benefit from de-implementation [25].

In 2000, Palit et al. returned to the question of the costs of breast-conserving surgery versus modified radical mastectomy, in light of the evolving nature of breast cancer treatment, especially the integration of post-mastectomy reconstructive surgery into treatment protocols [26]. They found that women who received breast-conserving surgery had a shorter length of stay and a lower cost of surgery than women who received modified radical mastectomy (with or without reconstruction) [26]. However, the addition of radiotherapy (typically 25 daily sessions) made the total cost of breast-conserving surgery greater than that of modified radical mastectomy alone and 16% greater than that of modified radical mastectomy with reconstruction [26]. Palit et al. found that breast-conserving surgery was underutilized with respect to the number of women eligible and observed that the preference for modified radical mastectomy “appears to be guided primarily by physician attitudes and confusion regarding standard eligibility criteria” ([26]: page 444). Therefore, from an economic perspective, it appears that modified radical mastectomy would be difficult to sustain were it not for the positioning of the surgeon, who performs the biopsy that confirms the diagnosis and tumor staging, and thereby has the benefit of a series of ongoing contacts with the woman patient and therefore the opportunity to exert influence in the choice of breast cancer treatment.

Furthermore, if a government payer or a private insurance company continues to reimburse a drug, device, or procedure—even in the face of an evidence base that indicates the need for de-implementation, most likely there will be little incentive for de-implementation.

### The context of politics and specialties

Some drugs, devices, and procedures have a well-established association with a particular practice specialty. Those seeking to promote the de-implementation of a technology must consider who “owns” the drug, device, or procedure and assume that those clinicians would want to keep what they have.

With respect to radical mastectomy, surgeons, who historically sought to distinguish themselves from generalists who could only offer palliative care, were the specialists who offered a treatment. After perfecting the procedure of radical mastectomy, it became the standard treatment for breast cancer. Paradoxically, this routinization of a procedure in order to reduce uncertainty about when to use it resulted in “deskilling” or loss of clinical skills or judgment. Surgeons, in fact, were left with one approach to all permutations of breast cancer. If the surgery failed to stop the disease, treatment failure was attributed to the surgery coming too late or being too conservative [27]. Without diagnostic procedures or alternative therapies, radical mastectomy became entrenched as the only treatment for breast cancer, and surgeons were the specialists who treated it.

Radical mastectomy reached its heyday in the USA during the 1950s, a time when radiation oncology was beginning to take hold in Europe [15]. Advances in diagnostic radiology resulted in mammograms that provided early detection, making it increasingly untenable for surgeons to continue to defend radical mastectomy as the standard treatment when faced with patients who had small tumors and a degree of malignancy that could now be staged by ever more sophisticated pathology analysis. Furthermore, by the late 1970s, surgeons were doing biopsies on an outpatient basis, and women patients were demanding a “two-step” procedure, i.e., separating diagnostic biopsy from breast cancer treatment. Biopsy allowed surgeons to hold their position as the point of first contact in the definitive diagnosis of breast cancer, but once through the gates and armed with a pathology report, women patients with early non-metastasized cancer could seek the treatment options that involved other specialties.

### Social context

De-implementation does not necessarily occur within the protected confines of research science and clinical practice. Research and practice are situated in a broader social context, and under some circumstances, the timing and sequencing of developing, assessing, adopting, and abandoning drugs, devices, and procedures are subject to outside social forces that affect health services.

The interest in de-implementing radical mastectomy extended beyond biomedical researchers and clinicians. At the same time that an evidence base was developing in the USA, the social movements of the 1970s and 1980s catalyzed the women’s health movement. Movement participants challenged the authoritarian relations within the practice of medicine, insisted on patient participation in medical decision-making, recognized patients’ psychological and social needs, and advocated that the patient’s experience be acknowledged in the doctor-patient encounter. Lay activists encouraged women with breast cancer to seek consultation from radiation and medical oncologists and diagnostic biopsies from surgeons with a reputation for considering the possibility of lesser surgery.

This sentiment was reflected in popular articles indexed in the *Readers’ Guide to Periodical Literature* [28] that discussed radical mastectomy. These articles were published in high-circulation journals, such as *Readers’ Digest* (17,829,000), *McCall’s* (7,500,000), *Good Housekeeping* (5,600,000), *Newsweek* (3,000,000), *Consumer Reports* (2,150,000), and *Glamour* (1,946,078) [29]. The intensity of the viewpoints expressed is indicated by these select titles of some articles: “Breast Cancer Debate: Mastectomy study” [30], “Controversy over breast cancer: Radical mastectomy” [31], “Right to choose: Mastectomy?” [32], “Breast cancer: The retreat from radical surgery” [33], “I said no to my doctors” [34], and “Breast Cancer: Death to the Radical?” [35]. Furthermore, journalists often interviewed critics of radical mastectomy, such as surgeon George Crile Jr. [36], noted coordinator of the national randomized controlled trial of breast cancer treatments Bernard Fisher [37], and coordinator of cross-national trials Gianni Bonadonna [38].

The reporting on breast cancer in the popular literature indicates that consumers were aware that there was controversy within medicine and that there were treatment alternatives to radical mastectomy. This awareness may have led to some degree of patient pressure on surgeons. In a 1985 television interview on lumpectomy, surgeon Susan Love claimed that, “This treatment option was not developed by doctors and surgeons looking for a better way. It was women who said, ‘I refuse mastectomy. You better find another way to treat me’” [39]. De-implementation came at a time when there was a convergence in clinicians’ and patients’ awareness of the scientific evidence, patients expressing their preferences for an alternative, and when there were other viable treatments to offer instead of radical mastectomy.

While changing social forces strengthened women patients’ role in de-implementing radical mastectomy, recently researchers have noted an upward trend in the rate of mastectomy. Katz et al. reported the relationship between patients’ retrospective reporting of their involvement in breast cancer treatment decision-making and the actual treatment they received [40]. Their survey respondents reported that their concerns regarding the recurrence of disease and the effects of radiation gave rise to their preference for mastectomy over lumpectomy [40]. Gomez et al. found increasing rates of mastectomy in women diagnosed with ductal carcinoma *in situ* (DCIS) who were white, younger than 50 years at diagnosis, and living in the highest 20% of neighborhoods ranked by socioeconomic status [41]. The authors contended that this group of women, who we would expect to have high decision-making agency, most likely preferred mastectomy because of negative attitudes toward radiotherapy, positive expectations regarding reconstruction, their understanding of their risk of recurrence, and not wanting to undergo tamoxifen therapy for 5 years post-lumpectomy (as recommended by a 2009 practice guideline, tamoxifen therapy was not necessary for women who were treated by mastectomy [42]). While we laud these researchers for considering the patient’s role in treatment decision-making, we note that their methodology did not employ direct observations of the physician-patient discussions of treatment courses (such as can be found in *Routine Complications* by socio-linguist Candace West [43]). Therefore, it is possible that surgeons influence their patients’ knowledge, attitudes, and beliefs regarding the risk of recurrence of breast cancer, the possible effects of radiation, the utilization of tamoxifen, and the promise of plastic surgery reconstruction. Given that a modified radical mastectomy is a quicker and easier surgery than breast-conserving surgery that in the USA commands higher surgeon fees [26], and is followed by subsequent reconstructive surgery that upper socioeconomic status women would be able to afford, a consideration of financial and professional forces would suggest that surgeons may be offering guidance that favors mastectomy over lumpectomy [26].

In Canada, where the costs of cancer surgery, radiation, and chemotherapy treatment are covered by provincially funded healthcare plans and provided to all citizens and landed immigrants, surgeons receive the same fee for either breast-conserving surgery or mastectomy (modified radical or otherwise). Plastic surgeons are the sole beneficiaries of a woman’s choice of reconstruction after a modified radical mastectomy. A recent Canadian Institute for Health Information (CIHI) report found that mastectomy rates exceeded 50% for women who would have to travel 1.5 h or longer (each way) to reach a center offering radiotherapy [44]. In this case, it seems plausible to assume that women who live in rural or sub-rural areas are avoiding travel to the approximately 25 daily radiation therapy visits following breast-conserving surgery, rather than expressing a preference for modified radical mastectomy surgery. Thus, the distance a woman has to travel between her home and a cancer center is a care delivery structural feature that can be conceptualized as a social force that sustains the use of modified radical mastectomy.

### Summary

In sum, evidence did play a role in the case of de-implementing radical mastectomy—but it was not the star of the performance as would be hoped for from an evidence-based medicine perspective.

We share Prasad and Ioannidis’ ideals regarding the evolution of ever-better science to guide implementation and de-implementation [7]. However, there are many cases of factors other than science catalyzing and sustaining misguided implementation and of sound and proven science failing to catalyze de-implementation [27,45-47]. Even when the empirical evidence is strong, scientific arenas may not necessarily be where the implementation/de-implementation decisions are made. Most scientists eschew advocacy, preferring to “let the science speak for itself” [48]. However, how can the science be heard, when there is a great deal of clatter from extra-scientific historical, economic, professional, and social forces in the practice arena?

Traditionally, the approach used to try to keep clinicians up to date has been continuing professional development or continuing medical education that relies on pedagogical theory. This underlying assumption of this approach is that if you educate people, they will be enlightened and will change their actions in response to the knowledge gained. In our experience, telling clinicians that the scientific evidence says what they are currently doing is no longer beneficial may not convince them to de-implement because it may not have been the scientific evidence that established the practice nor sustained its utilization [2].

In the field of implementation science, the role of psychological theories in understanding and predicting clinician behavior is influential. This should not be surprising given that ultimately individual clinicians (and their patients) make and follow through (or not) on decisions about care. While we acknowledge that these theories are helping to build our understanding of de-implementation, we believe that psychological theories are best utilized in the interpersonal realms of how we recruit, mobilize, involve, and retain clinicians on the teams that work for de-implementation. For example, Bowen and Graham have proposed an *Engagement Paradigm* in which scientists and clinicians collaborate to create evidence that is pre-designed to be clinically relevant and utilizable [49]. Furthermore, Ong et al. demonstrated that even when trying to precipitate behavior change (a key issue for psychology), psychological theory may offer little assistance [50]. Instead, they analyzed patient practices and strategies in the broader social context to explain why, for whom, and under what circumstances a treatment works [50].

To advance the science of de-implementation, we argue that the time is ripe for more in-depth analysis and understanding of all of the factors operating to sustain commitment to medications, devices, procedures, interventions, and tests that the evidence slates for de-implementation. There is a wealth of work from the disciplines of *Science and technology studies*, the *History and philosophy of science*, the *Sociology of health and illness*, and *Medical Anthropology* that addresses issues of de-implementation in medicine and beyond. Table 1 delineates these disciplines, what they study, issues commonly addressed, methodological approaches typically taken, and a sample publication for each.

**Table 1:** **Disciplines that address de-implementation in medicine and the contexts of clinical practice**

**Discipline**	**What is studied**	**Some issues of studies**	**Some approaches**	**Sample publication**
Medical anthropology	The ways in which culture and society are organized around or influenced by issues of health, health care and related issues	Folk medicine Ethnobotanical knowledge The culture limits of biomedicine	Field research Participant observation	Aggarwal NK, Nicasio AV, DeSilva R, Boiler M, Lewis-Fernández R. “Barriers to implementing the DSM-5 cultural formulation interview: a qualitative study.” *Cult Med Psychiatry.* 2013 Sep;37(3):505–33 [51]
Science and technology studies	How social, political, and cultural values affect scientific research and technological innovation	Biotechnology Environmental sustainability Information technology	Science citation index analysis Historical analysis Case comparisons	Obstfelder A, Engeseth KH, Wynn R. “Characteristics of successfully implemented telemedical applications.” *Implement Sci.* 2007 Jul 27;2:25 [52]
Sociology of health and illness	Medical organizations and institutions, the production of knowledge and selection of methods, the actions and interactions of healthcare professionals, and the social or cultural (rather than clinical or bodily) effects of medical practice	Experiences of patients Health disparities Interactions between sick people and healthcare practitioners	Qualitative interviewing Demographic analysis Survey research	Timmermans S, Berg M. “The practice of medical technology.” *Sociol Health Ilnn.* 2003;25:97–114 [53]
History and philosophy of science	Science, its nature and fundamentals, its origins, and its place in modern politics, culture, and society	How the sciences originated, how they were practiced, how they were developed, and how they were related to their intellectual and social contexts	Archival research Textual analysis Re-enactment of experiments	Richard W,. Wertz and Dorothy C. Wertz. *Lying-In: A History of Childbirth in America.* 1989, Yale University Press [54]

There is much to be learned from applying social science approaches to study both resistance to de-implementation (e.g., radical mastectomy [27] and episiotomy [46]), as well as appropriate and even rapid de-implementation (e.g., halting the prescribing of *rofecoxib* and hormone replacement therapy). Contrasting these findings with the results from studies of rapid (both appropriate and inappropriate) implementation (e.g., the rapid adoption of experimental HIV drugs [45]) will advance our understanding of de-implementation. Furthermore, as we consider the extra-scientific forces that sustain “entrenched practices and other biases” [7], we need to search literature not indexed in typical medical databases such as PubMed, CINAHL, and EMBASE for socio-historical analyses of de-implementation in health and other fields (see for example, a provocative study of CT scanners in radiology departments published in the management literature [55]).

In sum, we contend that even when clinicians know what practices are in accord with scientific evidence, they often remain confined by a set of structural forces beyond their control and are not able to make changes. We propose to conceptualize de-implementation as systems change, leading to work for de-implementation at the systems level. We suggest intervening in the broader context in which clinicians work—the social, political, and economic realms—to change systems rather than trying to change individuals. We also argue that the time is right to further develop our de-implementation theories to incorporate the social science that considers historical, economic, professional, and social forces for change.
